# Management of Penetrating Thoracoabdominal Trauma in a Resource-Limited Setting: A Case Report

**DOI:** 10.7759/cureus.106998

**Published:** 2026-04-13

**Authors:** Zac W Riggenbach, Brooklyn Williams, Jonathan Lutgens, Abigail Kelly, Alexander Malloy

**Affiliations:** 1 General Surgery, Madigan Army Medical Center, Tacoma, USA; 2 Trauma and Acute Care Surgery, Harborview Medical Center, Seattle, USA

**Keywords:** global health surgery, impalement injury, resource-limited settings, thoracoabdominal trauma, trauma surgery

## Abstract

Trauma remains a major global health challenge, particularly in low- and middle-income countries where access to advanced surgical resources is limited. This case report describes the management of penetrating thoracoabdominal impalement in a resource-constrained hospital in Honduras during a Medical Readiness Training Exercise (MEDRTE). An adult male presented after being impaled by a metal spike through the left thoracoabdominal region. With limited imaging and no access to blood products, the patient underwent emergent exploratory laparotomy and thoracotomy. Injuries included gastric perforation, splenic laceration, diaphragmatic injury, pulmonary laceration, and a pericardial tear. The patient recovered without complications and was discharged on postoperative day 5. This case highlights the challenges of trauma care in austere settings and underscores the importance of surgical adaptability and real-world training experiences such as MEDRTE deployments.

## Introduction

The surgical management of traumatic injuries has advanced significantly over the past several decades, driven in large part by military conflicts, such as new resuscitation practices, tourniquets, and patient transport goals [1]. Recent advancements - such as the implementation of Advanced Trauma Life Support (ATLS) protocols and improvements in trauma resuscitation with balanced blood product administration - have markedly improved mortality outcomes in both civilian and military populations [2]. Despite this progress, resource-limited environments often do not have access to these new treatment advances and require thoughtful management to provide the best care.

The surgical management of trauma in resource-constrained environments is of increasing importance, especially as ongoing global conflicts continue to create austere and unstable conditions. In such contexts, surgeons are often required to perform complex procedures with limited supplies, infrastructure, and support. This case report details the management of a patient with extensive thoracoabdominal trauma in a resource-limited hospital in Honduras. Limitations present in this case included a lack of access to advanced imaging, a lack of blood products for resuscitation, and access to surgical subspecialists, among other challenges. These limitations necessitated clinical management that differs from standard-of-care practices in most high-resource medical centers and the decision-making process involved in those deviations. It provides a real-world example of trauma care in austere conditions and highlights the principles and skills necessary for effective surgical intervention under such constraints. This case can be used for further discussions on appropriate training and development plans for surgeons who aspire to provide care in resource-limited and austere conditions.

## Case presentation

In early 2019, a group of uniformed military personnel participated in a Medical Readiness Training Exercise (MEDRTE) in Honduras. The mission involved providing both hands-on medical support to the local healthcare system and conducting training to enhance surgical and trauma care readiness. At the host hospital, the available resources were extremely limited: there was no formal ATLS training among the staff, no organized trauma care program, no access to blood products, and no trauma surgeon on site. Mechanical ventilators and other advanced medical equipment were brought by the MEDRTE team as part of their outload kit.

**Figure 1:**
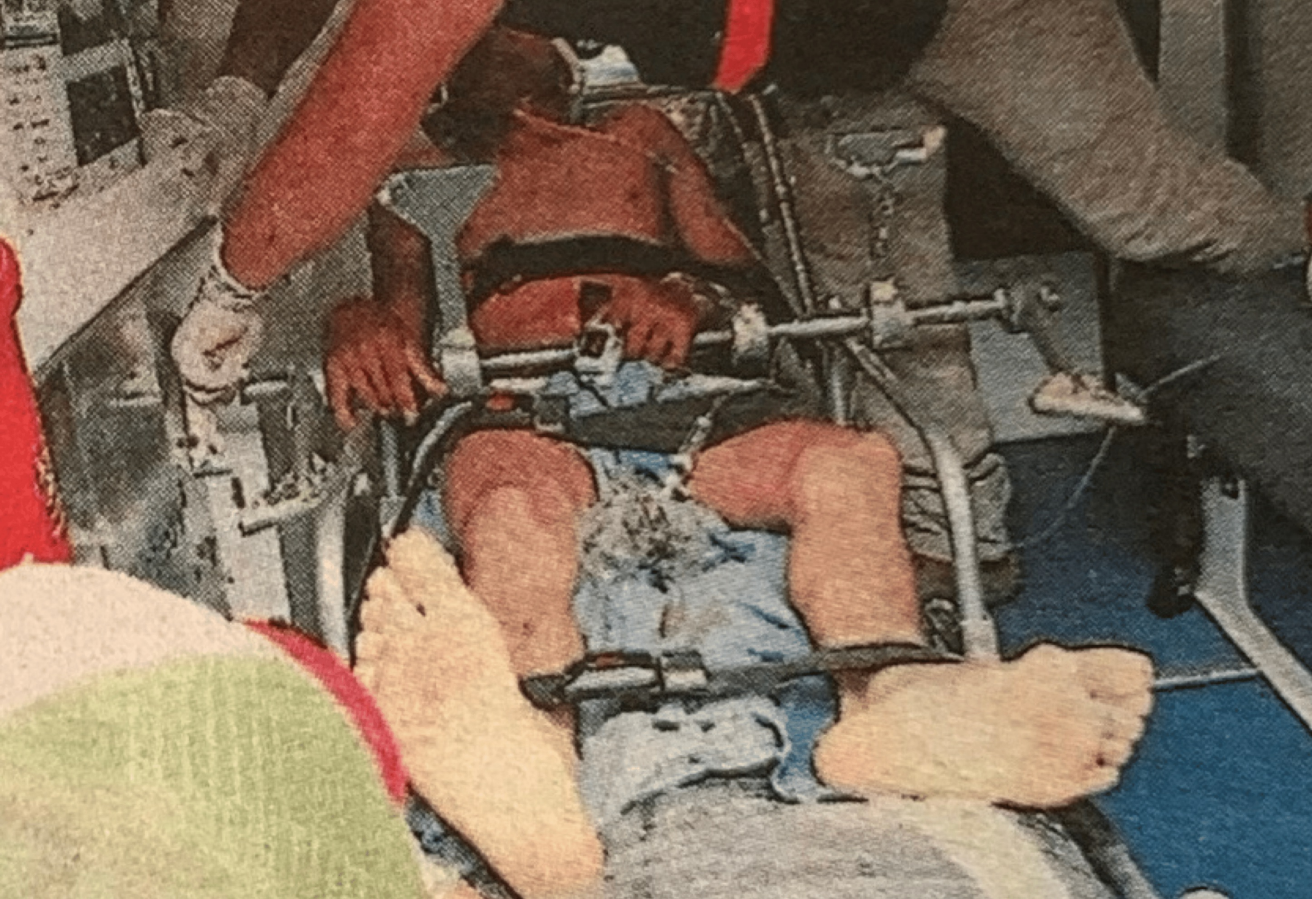
This figure is an image taken during the on-scene response from local EMS teams. Note the metal spike impaled in his left hemi-abdomen.

During the deployment, an adult male presented to the hospital after sustaining significant thoracoabdominal trauma at his workplace. The patient had been working in a food processing facility when he was impaled through the left thoracoabdominal region by a metal spike, as shown in Figure 1. Emergency transport brought him to the local hospital for surgical evaluation and management with the machinery left in situ. The patient arrived at the medical facility within 1 hour of his initial injury.

**Figure 2:**
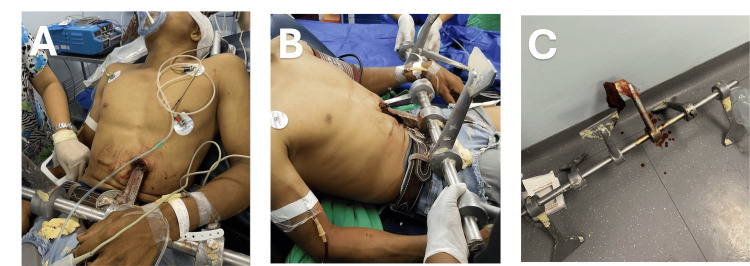
This figure shows images from the patient's initial presentation to the local Emergency Department. Image 2A shows the spike still impaled into the patient from an anterior perspective. Note the initial entry point in the left upper quadrant of the abdomen. Image 2B again shows the initial impalement trajectory from a lateral perspective. Notice the upward trajectory into the thorax. Image 2C shows a close image of the spike after it has been delivered from the abdomen.

On presentation, the patient was noted to be initially hypoxic, which improved to oxygen saturations >92% on a non-rebreather mask and supplemental oxygen. He was otherwise hemodynamically normal. His shock index remained <0.9 for the entirety of his initial transport and primary survey. A rapid physical examination and plain chest-abdominal radiograph demonstrated a single metallic impalement entering the left upper quadrant of the abdomen and tracking superiorly toward the thorax. With no access to a tertiary care trauma facility, no access to blood products, and limited diagnostic imaging capabilities, the patient received an initial resuscitation with 2L of crystalloid and was immediately taken to the operating theatre for surgical exploration within minutes of his arrival to the medical center. Photos from his initial evaluation in the operating room are shown in Figure 2.

The operative team consisted of a fellowship-trained Trauma and Critical Care Surgeon and a Certified Registered Nurse Anesthetist (CRNA) from the MEDRTE team, in coordination with a local General Surgeon and operating room staff. Due to the hospital’s lack of functional ventilators, mechanical ventilation during surgery was supported by the portable ventilator supplied by the MEDRTE unit. Similarly, no invasive blood pressure monitoring devices were available during the procedure. Instead, his resuscitation and intraoperative monitoring were supported primarily by non-invasive blood pressure monitoring, pulse oximetry, and clinical gestalt.

**Figure 3:**
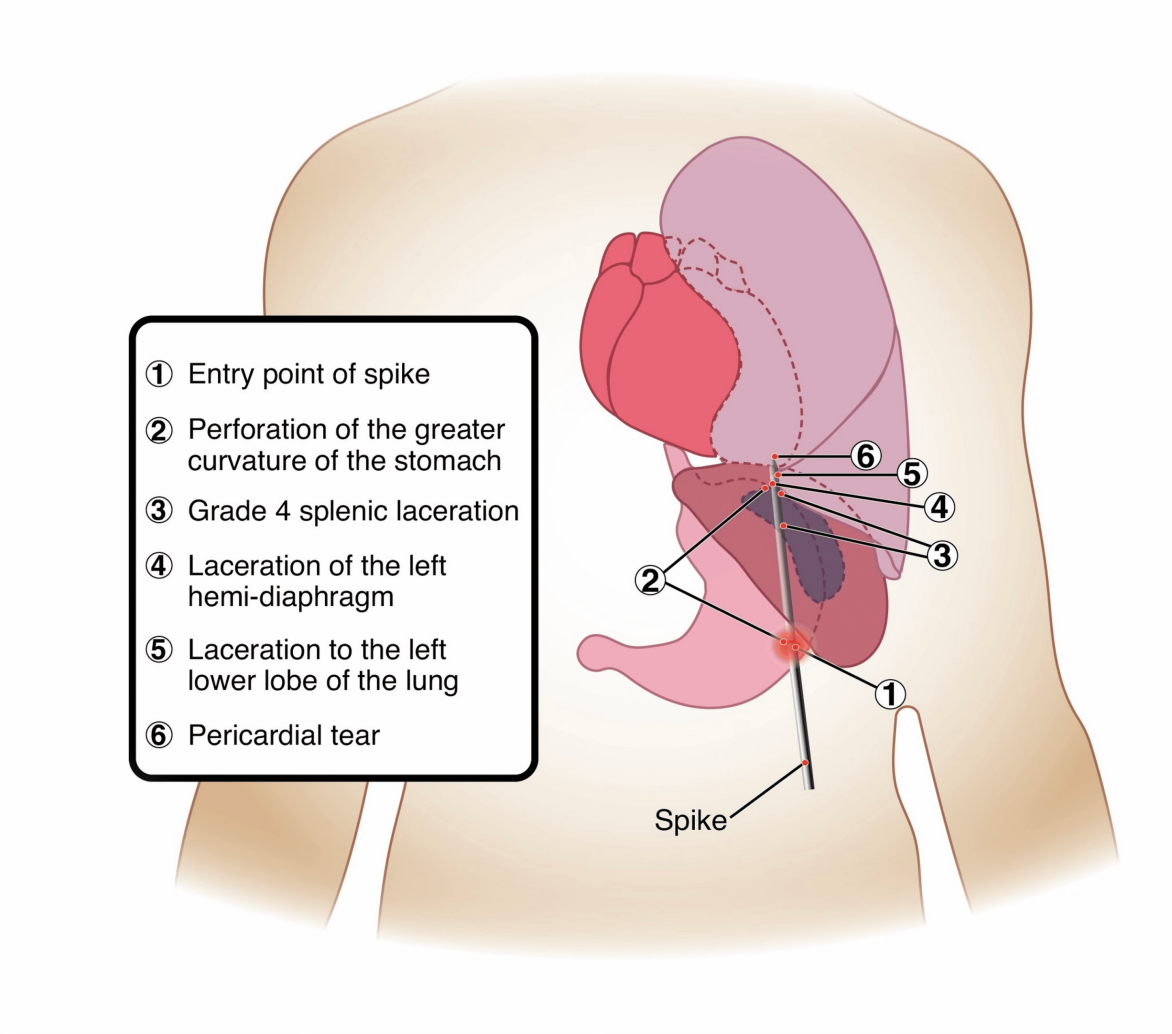
This illustration offers a representation of the impalement of the spike and its trajectory. Notice the entry location in the left upper quadrant of the abdomen with a superior trajectory into the left thorax. Additionally, each of the major injuries are listed, shown and labeled. Image credit: Harper D’Angela, using Adobe Illustrator (Adobe Inc., San Jose, California, USA).

The patient presented with a complex wound pattern - an impaling thoracoabdominal wound with a retained foreign body - which required exploration of both the abdomen and thorax. Given the complexity of the wound and two available surgeons, the operative team decided to attempt a simultaneous exploration of both the thorax and abdomen. In the operating room, the Trauma Surgeon performed a midline exploratory laparotomy while the General Surgeon performed a left anterolateral thoracotomy. Upon entering the peritoneal cavity, approximately 1 liter of hemoperitoneum was encountered. Exploration revealed a perforation along the greater curvature of the stomach, an AAST grade 4 splenic laceration, and a laceration of the left hemidiaphragm. Simultaneously, thoracic exploration uncovered a laceration to the left lower lobe of the lung and a pericardial tear without evidence of myocardial injury or tamponade. An illustration showing his traumatic injuries is shown in Figure 3.

Initial hemorrhage control was achieved via manual packing. Following this, the metallic mixing spike was carefully withdrawn along its entry path - from the thoracic cavity through the diaphragm and abdomen - under direct visualization to prevent secondary injuries. The surgical team then divided responsibilities: the Trauma Surgeon addressed the intra-abdominal injuries while the local General Surgeon continued managing the thoracic injuries. A splenectomy was performed using clamps and sutures. Due to the size and location of the gastric perforation, a primary repair was not feasible. The gastric injury was repaired using a series of linear staple fires medial to the gastric laceration along the greater curve of the stomach. This staple line was able to successfully exclude the defect in the gastric body without narrowing the stomach. The excluded stomach was completely divided from the stomach via the staple line and removed from the abdomen. The diaphragmatic laceration was repaired primarily with 0 Ethibond suture. A complete evaluation of the bowel and remaining abdominal organs did not reveal any additional injuries. The abdominal cavity was irrigated, and the wall was closed in a layered fashion using 0 PDS sutures. A surgical drain was placed in the splenic bed prior to closure.

Concurrently, the thoracic team performed a wedge resection of the injured portion of the left lower lung lobe using surgical staplers provided by the MEDRTE team. The pericardial tear was extended to allow complete evaluation of the myocardium, which was found to be intact. The pericardium was then loosely reapproximated to mitigate the risk of postoperative tamponade. Two 32 French chest tubes were placed for postoperative drainage. Total blood loss during the case - including the estimated 1 L hemoperitoneum from initial entry - was estimated at 1.5 L.

The patient was hemodynamically normal during his operation and remained so during his hospital recovery. He was admitted to the hospital floor, and he was slowly advanced to a regular diet by postoperative day 4. The abdominal drain was removed on postoperative day 3, and chest tubes were removed on days 3 and 4. The patient was discharged on postoperative day 5. Post-splenectomy vaccination status was unclear as these vaccines were not immediately available at the local facility. At follow-up, he had returned to his baseline activity level without any observable long-term impairments.

## Discussion

This case exemplifies a dramatic instance of thoracoabdominal impalement managed in a resource-constrained environment where transfer to a tertiary care facility was not feasible. Unfortunately, trauma is one of the most rapidly growing and deadly global health challenges, surpassing even HIV/AIDS in mortality [2]. Its impact is even more severe in low- and middle-income countries (LMICs), which carry a disproportionate share of the trauma burden, as seen in this case [2].

To better understand the challenges in providing high-quality trauma care in LMICs, frameworks traditionally used in maternal health, such as the “three delays” model, have recently been applied: (1) delay in seeking care, (2) delay in reaching care, and (3) delay in receiving appropriate care [3]. Understandably, Delay 3 has received much of the attention in research and represents a key focus when considering the challenges of LMICs trauma care [3]. In this case, the third delay - access to definitive care - was again the most significant barrier. The absence of essential resources such as blood products, advanced diagnostic imaging, and comprehensive trauma systems meant that successful patient outcomes depended heavily on the adaptability, technical breadth, and coordination of the surgical team.

For example, resuscitation of trauma patients has undergone substantial evolution over the past two decades, influenced heavily by combat casualty care during the wars in Iraq and Afghanistan [1]. Modern trauma centers now employ balanced transfusion strategies with whole blood or component therapy, which have demonstrated significant survival benefits even in austere military contexts [1]. Unfortunately, such advancements remain out of reach in many LMICs, where blood banking infrastructure and cold chain capabilities are frequently absent. Although this patient was hemodynamically stable on arrival, the lack of blood products posed a considerable risk in the setting of known intra-abdominal hemorrhage, where early transfusion would have been standard in a high-income country (HIC) context. In this case, an initial resuscitation with 2L of crystalloid was deemed appropriate to support his suspected intra-vascular volume depletion. However, these decisions must be taken with caution as further resuscitation would risk a compounding dilutional coagulopathy [4].

The lack of advanced imaging, particularly computed tomography (CT), further complicated care. While CT scanning may not have altered the surgical management in this particular case, the absence of diagnostic tools illustrates a broader limitation in LMIC trauma systems. In HICs, imaging and interventional radiology play a critical role in triaging stable patients, identifying occult injuries, and planning operative approaches [5]. The increased utilization of these devices and strategies has led to an increasingly high percentage of non-operative management of traumatic injuries [5]. Similarly, the now common Focused Assessment with Sonography in Trauma (FAST) exam, which often helps direct the decision for expeditious operative management in trauma patients, was not available to assist in the decision-making process for this patient [6]. In many HIC environments, this patient may have received additional imaging, such as CT or FAST, to confirm intra-abdominal penetration and the extent of his thoracic injuries. Unfortunately, those modalities were not available in this setting. Resource-limited settings like the one demonstrated here, therefore, often require reliance on clinical judgment and exploratory surgery - an approach that can be both life-saving and unnecessarily invasive. In this case, the penetrating nature of his wounds within his abdomen and the retained body necessitated operative intervention. Further evaluation in the limited emergency department, without any advanced imaging or laboratory capabilities, would not have improved his care and only delayed his needed operative management. As such, early operative intervention in this patient’s case was preferable despite the fact that he may have received other interventions in an HIC setting.

Beyond his initial management, the LMIC setting further complicated his post-trauma care. Post-splenectomy vaccines are considered standard-of-care in most HIC [7]. However, this patient’s vaccine status remains unclear due to the lack of availability at the local hospital. This unfortunately reflects an overall poor access to these vaccines in LMIC [7]. This case, therefore, highlights another key difference in the management of trauma patients in LMICs. While this patient clinically required a splenectomy in this situation, surgeons operating in austere environments or LMIC require a deep understanding of the local medical infrastructure to make the best decisions for what will likely result in complex, long-term health care decisions.

While these limitations seen in this case present clinical challenges, they also serve as a unique and valuable training opportunity for surgeons preparing to work in austere conditions, including combat zones and disaster areas. Historically, surgeons have played essential roles in both civilian and military responses to conflict, but most are trained in well-resourced academic environments that do not adequately reflect the logistical and clinical realities of war zones [8-10]. The gap between conventional training and the realities of field surgery is particularly concerning, given recent geopolitical instability. Conflicts in Ukraine, Israel, and Iran suggest a potential return to large-scale combat operations, where surgeons may be deployed to settings with little support infrastructure [11-13].

The Medical Readiness Training Exercise (MEDRTE) program, during service from which this case originated, stands out as a rare opportunity for hands-on surgical experience in low-resource settings. By embedding military healthcare providers within LMIC medical systems, these exercises expose participants to real-world challenges, including limited supplies, reduced staff availability, and the need for improvisational decision-making. As demonstrated in this case, such programs not only offer critical support to host nations but also provide immersive, practical training that cannot be achieved through simulation alone [8]. This hybrid model of humanitarian assistance and medical education may represent one of the most effective strategies for preparing healthcare personnel for future deployments to austere or conflict-affected regions.

## Conclusions

This case demonstrates the management of complex, penetrating thoracoabdominal trauma in a severely resource-limited setting. It underscores the practical challenges faced by surgeons operating in LMICs and highlights the necessity of versatile surgical skills, adaptability, and creative problem-solving. It also highlights the importance of understanding local medical infrastructure, such as access to post-trauma care and vaccine availability, by the surgeon. Moreover, it offers an example of real-world training experiences such as those provided by the MEDRTE program. As global instability continues, it is imperative that surgical training and readiness evolve to ensure that providers are equipped to deliver high-quality care in austere environments.
